# *Streptococcus pneumoniae* upregulates *Toll2*, *Toll9*, and *defensin* genes in *Bombyx* larvae infection model

**DOI:** 10.1371/journal.pone.0341929

**Published:** 2026-01-30

**Authors:** Farhan R. Chowdhury, M. Ismail Hossain, Tangerul A. Jepu, Nusrat U. A. Saleh, Fatema T. Zohora, Tasmim A. Saleh, Mrinmoy Sarker, Al Numan, Zainab Yousuf, M. Aftab Uddin, Muktadir S. Hossain

**Affiliations:** 1 Department of Biochemistry and Microbiology, School of Health and Life Sciences, North South University, Dhaka, Bangladesh; 2 Bangladesh Sericulture Research and Training Institute, Rajshahi, Bangladesh; 3 Core Research Facility, School of Health and Life Sciences, North South University, Dhaka, Bangladesh; Okayama University, JAPAN

## Abstract

Pneumococcal diseases caused by the human pathogenic bacterium *Streptococcus pneumoniae* are a major public health concern worldwide. In this study, we examined the pathogenicity of a clinical isolate of *S. pneumoniae* in the silk moth, *Bombyx mori*, larvae infection model. The whole genome sequencing of a clinical isolate of *S. pneumoniae*, Spn1 identified the presence of genes responsible for its virulence and antibiotic resistance. Spn1 infection of *Bombyx* larvae resulted in death within 24 h concomitant with an increase of phenoloxidase activity in the hemolymph. The bacterial load increased in the hemolymph within 9 h post-infection (p.i.) Ampicillin, ceftriaxone, tetracycline, imipenem, and erythromycin showed therapeutic effect in infected larvae, although the bacterial strain was resistant to erythromycin *in vitro*. The *Bombyx* homologs of mammalian *TLR2* and *TLR4*, known as *BmToll2* and *BmToll9* (*BmToll9−1* and *BmToll9−2* isoforms), were upregulated in both the fat body and trachea. The antimicrobial peptide (AMP) genes, *BmdefensinA* and *BmdefensinB*, known to be regulated by the Toll signaling pathway, were significantly upregulated in both fat body and trachea after *S. pneumoniae* infection through hemolymph. Our data indicate that the *Bombyx* larvae can be a suitable infection model to study the pathogenicity of *S. pneumoniae*.

## Introduction

*Streptococcus pneumoniae*, known as the pneumococcus, is a Gram-positive, facultative anaerobic, pathogenic bacterium primarily responsible for causing community-acquired pneumonia and meningitis in children under five years old, as well as in elderly people and those with pre-existing health issues. It is estimated that about one million children die worldwide of pneumococcal disease every year, most of whom are young children in developing countries [1].

*S. pneumoniae* is commonly found in normal flora of the upper respiratory tract, typically colonizing the respiratory tract, sinuses, and nasal cavity [2]. It is a significant bacterial agent responsible for various diseases, including otitis media, sepsis, and sinusitis, as well as community-acquired pneumonia and meningitis [3]. This condition is the predominant cause of complicated pneumonia in pediatric populations [4,5]. The use of antibiotics or vaccines is the two main methods to prevent *S. pneumoniae* infection. Although vaccination against *S. pneumoniae* can be an effective way to prevent pneumococcal infection, antibiotics are essential in reducing bacterial load [6]. *S. pneumoniae* has a remarkable capacity to acquire exogenous DNA from other pneumococci or related streptococci to become antibiotic-resistant and evade vaccine-induced immunity [7]. The unregulated prescription of antibiotics, self-administration of antibiotics, inadequate hygiene in countries with low- and middle-income, and excessive use of antibiotics in agriculture are all factors contributing to the rise of antibiotic-resistant strains [8,9].

Before being used in human trials, novel antibiotics should be tested for their effectiveness and possible adverse effects in a suitable animal model. The utilization of prevalent mammalian model species, like mice, in comprehensive studies aimed at screening for novel antibiotics that can combat newly emerging antibiotic-resistant bacterial strains presents significant financial challenges. There are also ethical considerations linked to the use of mammalian organisms. The *Bombyx* larvae have been used as an infection model for microorganisms pathogenic to humans [10,11]. The *Bombyx* larvae infection model is a useful to study not only host-pathogen interaction but also the therapeutic effect of antibiotics on a range of bacterial classes, including *Staphylococcus aureus*, *Escherichia coli* O157:H7, *Klebsiella pneumoniae*, *Niallia circulans,* and *Klebsiella aerogenes* [12–16]. This infection model has also been used to study fungal pathogenicity [17]. The prospect for discovering novel drugs effective against antimicrobial resistant (AMR) bacteria is also high according to the recent reports [18]. The fact that *Bombyx* larvae and mammals have comparable pharmacokinetics of antimicrobial agents makes it an attractive infection model to screen for antibiotics against bacterial pathogens [19].

Although insects apparently lack adaptive immunity, remarkable similarities have been observed with respect to immune response between insects and mammals [20,21]. Insect hemocytes or immune cells of blood recognize pathogens, phagocytose them, and kill the ingested microbes with superoxide production that is similar to neutrophils [22]. Even though there are some clear differences, there are a lot of similarities in how insects and humans encode the antimicrobial peptide (AMP) gene and how harmful bacteria turn on the Toll and Imd signaling pathways [23]. The Toll signaling pathway was first identified in the fruit fly, *Drosophila melanogaster*, which is essential for the dorso-ventral axis formation in the embryo [24], and later studies revealed its role in innate immunity, including activation of expression of AMP genes [25]. Around ten Toll-like receptors (TLRs) have been identified in mammals, and these transmembrane proteins act as pattern recognition receptors (PRRs) to detect the pathogen-associated molecular patterns (PAMPs) found on the surface of microbial pathogens [26–28]. These TLRs are important to fight off the invading microbes and prevent extensive tissue damage [29]. Among these TLRs, TLR2 and TLR4 primarily mediate the inflammatory responses upon infection by *S. pneumoniae* [30–32]. The lepidopteran insect, the silk moth, *Bombyx mori*, has 14 *Toll* genes, and among those, *BmToll2* and *BmToll9* are homologs of the mammalian TLR2 and TLR4 [33–35]. Two *Bombyx BmToll9* genes, *BmToll9−1* and *BmToll9−2*, have been identified recently [36,37].

Pathogenicity of *S. pneumoniae* has been reported in *D. melanogaster*, the tobacco horn moth, *Manduca sexta*, and the wax moth *Galleria mellonella* [38–40], however this study did not address the impact of the bacterium on the expression of homologs of mammalian TLR2 and TLR4 in these species. This research evaluated the efficacy of *Bombyx* larvae as an infection model for *S. pneumoniae*, aimed at both antibiotic screening and the examination of the evolutionary conservation of the Toll signaling system in the immune response to this significant human pathogen.

## Materials and methods

### Growth conditions of bacterial strains

In this research study, four clinical isolates of *S. pneumoniae* (labeled as Spn1, Spn2, Spn3, and Spn4) were used. The Spn1 strain was a kind gift from Dr. Samir K. Saha of Dhaka Shishu Hospital, and the other three strains were obtained from diagnostic centers of Dhaka, Bangladesh. Standard biochemical tests were conducted for Spn1 (S1 Table) and the other three strains. The AMR profile was established for Spn1 (S2 Table). Initially, the identity of the *S. pneumoniae* strains has been verified using 16*S* rRNA gene sequencing. *S. pneumoniae* strains were routinely grown on Brain Heart Infusion (BHI) agar supplemented with 5% defibrinated sheep blood at 37°C under aerobic conditions. For liquid culture, *S. pneumoniae* was cultivated aerobically at 37°C in PPB medium, composed of 20 g tryptone, 10 g yeast extract, 2 g glucose, 5 g NaCl, and 2.5 g Na_2_HPO_4_ per liter, with a pH of 7.5 as described previously [39].

### Whole-Genome Sequencing of Spn1 and bioinformatics analyses

To isolate genomic DNA from *S. pneumoniae* strain Spn1, the QIAamp® DNA Mini Kit (Qiagen, Hilden, Germany) was utilized according to the manufacturer’s guidelines. DNA quantity and quality were verified with Quantus Fluorometer (Promega) and NanoDrop 2000c (Thermo Fisher Scientific). Sequencing libraries were prepared with Illumina DNA prep kits, and paired-end sequencing was performed on an Illumina NextSeq 550 platform with a High Output Kit v2.5 (300 cycles). The raw sequencing reads were processed by trimming adapter sequences, and data quality was assessed using FastQC (v0.74) on the Galaxy Europe platform. The draft genome was assembled with SPAdes (v3.15.4), and functional annotation was performed using PROKKA (v1.14.6). The NCBI accession number of the draft genome of *S. pneumoniae* strain Spn1 is JBBMWB000000000. MLST typing was performed using the *Streptococcus pneumoniae* scheme implemented in the MLST tool (https://github.com/tseemann/mlst). The same typing scheme available at the Center for Genomic Epidemiology (CGE) was also used to confirm allele assignments. The genome was annotated with the RAST toolkit (RASTtk). Plasmid replicons and virulence genes were identified using PlasmidFinder 2.1 [41] and the Virulence Factor Database (VFDB) [42], respectively. Screening for antimicrobial resistance (AMR) genes was conducted using the Comprehensive Antibiotic Resistance Database (CARD) [43], AMRfinder [44], NCBI, and Resfinder [45]. PHASTER was used to identify prophages. A phylogenetic tree was constructed through TYGS using *S. pneumoniae* strain Spn1, employing the Generalized Time Reversible substitution model, with iTOL utilized for visualizing the tree [46]. To search for any CRISPR-Cas systems in the Spn1 genome, we used the CRISPRCasFinder tool [47], and the analysis was performed with default parameters on the web server.

### *B. mori* strain and rearing conditions

The Nistari-M strain of *B. mori* was reared at the Bangladesh Sericulture Research and Training Institute (BSRTI). Fresh mulberry leaves were fed thrice a day to the larvae, and they were kept at a temperature of 25 ± 1°C with 80% relative humidity and 16 h light: 8 h dark cycle. Experiments were conducted using larvae on the second or third day of the fifth instar.

### Bacterial injection, proliferation, hemocyte viability test, and phenoloxidase activity assay in *B. mori* larvae

For infecting larvae, an overnight culture of *S. pneumoniae* was used as described by our group previously [16]. Briefly, larvae (*n* = 10) were grouped together in boxes that were stored at 25 ± 1°C, 80% relative humidity and 16 h light: 8 h dark cycle. Feeding was stopped 2 hours before injection and the larvae are kept in fasting condition throughout the infection period. Larvae received injections of a specified quantity of bacteria suspended in PBS, delivered into an abdominal leg by means of a 30-gauge (30G × 5/16”). The number of proliferated bacteria in the hemolymph was determined through serial dilution and spread plating, and hemocyte viability was assessed using the Trypan Blue cell viability assay with a hemocytometer. Additionally, the hemolymph phenoloxidase (PO) activity assay was performed as previously described [15].

### MIC of antibiotics and ED_50_ determination in *Bombyx* larvae

To determine if *B. mori* larvae are a suitable infection model for testing antibiotic efficacy against *S. pneumoniae*, the following drugs were used: tetracycline (Sigma-Aldrich, USA), ceftriaxone (Radiant Pharmaceuticals, Bangladesh), ampicillin (Opsonin Dhaka, Bangladesh), imipenem-cilastatin (Incepta Pharmaceuticals Ltd., Bangladesh), and erythromycin (Square Pharmaceuticals Ltd., Bangladesh). Each antibiotic was diluted with distilled deionized water to four concentrations: 50, 5, 0.5, and 0.05 µg g ⁻ ¹ body weight. Larvae received a single 50 µL dose of antibiotic 10 minutes post-infection. No toxicity was observed for the drug-alone treatment at the highest doses of antibiotics used in the uninfected larvae. The minimum inhibitory concentration (MIC) of each antibiotic against *S. pneumoniae* was ascertained utilizing a Promega GloMax Explorer microplate reader, following Clinical and Laboratory Standards Institute (CLSI) criteria, as previously delineated [16]. The median effective dosage (ED₅₀) was determined using accepted methodologies [12].

### Topical application of *S. pneumoniae* on *Bombyx* larvae

To infect the silkworm trachea (airway system) through spiracles, we topically applied the indicated number of Spn1 bacteria suspended in 50 µL of PBS onto 10 abdominal spiracles (5 µL drop per spiracle across 5 spiracles on each side) using micropipette, whereas the control larvae were treated with PBS. Larvae were manually restrained without anesthesia during application and were kept isolated for 10 minutes before transferring to the boxes to prevent cross-contamination or oral exposure. RT-qPCR analysis was performed by isolation of RNA from tracheal bushes collected from dissected larvae after 6- and 12 h of infection. Bacterial numbers in hemolymph and tracheal bushes after topical application were determined as described previously [15].

### RT-qPCR analysis of *BmToll2*, *BmToll9−1*, *BmToll9−2*, and AMP genes

Gene expression analysis of adipose tissue and tracheal tissues was carried out through RT-qPCR, as previously published [15]. The total RNA was extracted from the fat body in the dorsolateral region of larvae or tracheal bushes from all over the body using TRIzol® (Invitrogen, USA) as per the manufacturer’s guidelines. cDNA synthesis was performed with the ProtoScript® II First Strand cDNA Synthesis Kit (New England Biolabs) following the provided protocols. RT-qPCR was carried out using the Luna® Universal qPCR Master Mix (New England Biolabs) on a Bio-Rad C1000 Touch™ Thermal Cycler. *Bmrp49* was used as the reference gene for normalization. Gene-specific primers for *BmToll2*, *BmToll9*−1, and *BmToll9*−2 were designed with Primer3 based on published sequences, and sequences of all the primers, including the ones for the antimicrobial peptide (AMP) genes, are listed in S3 Table. Gene expression was quantified using the 2^-ΔΔCT^ method.

## Results

### Identification of virulence genes in the genome of a clinical isolate of *S. pneumoniae* strain

**Fig 1:**
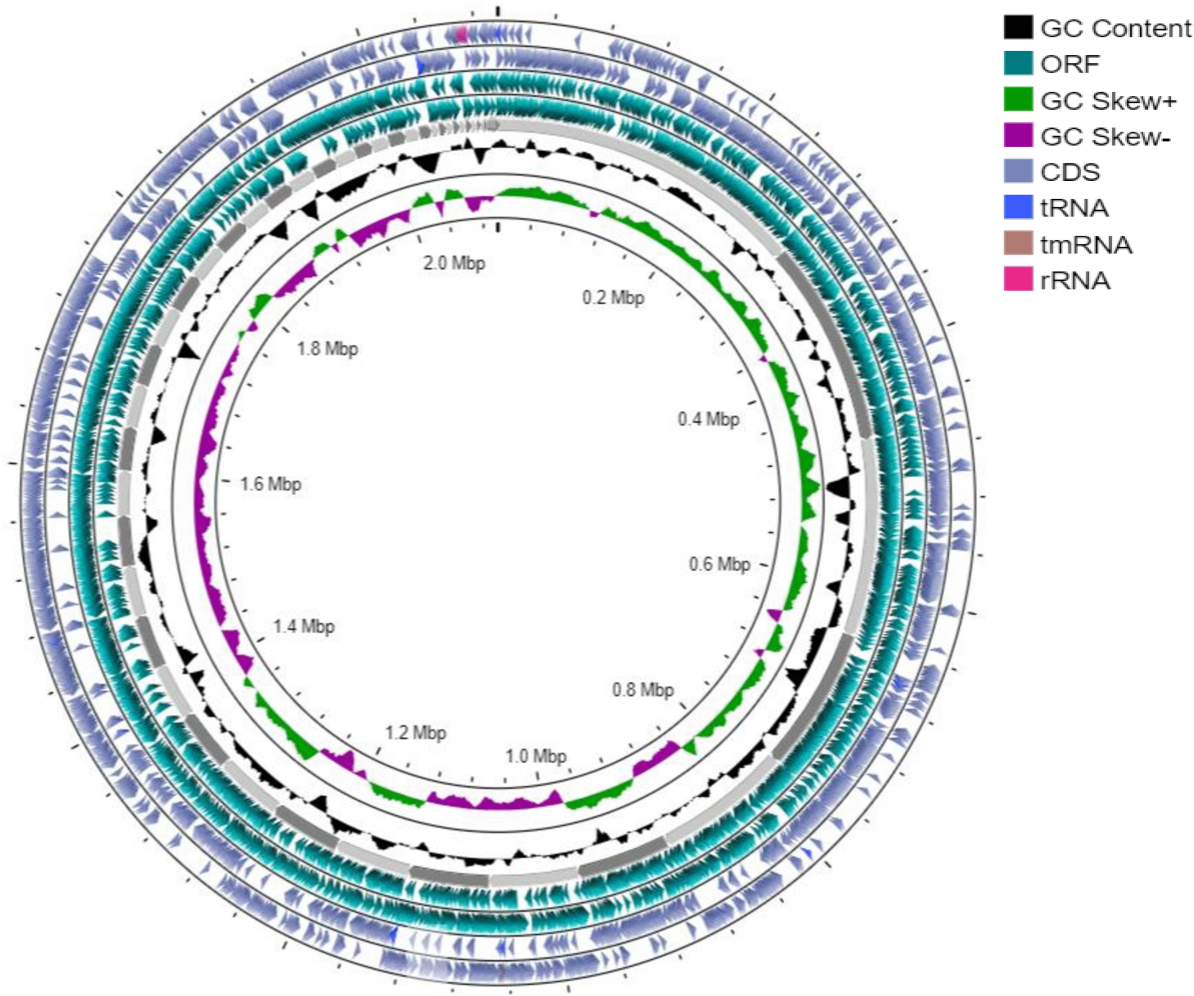
Maps showing the circular genome of *Streptococcus pneumoniae,* Spn1 chromosome. The contents of the feature rings (starting with the outermost ring) are as follows: Ring 1: reverse strand of coding sequence (CDS) features; Ring 2: forward strand of coding sequence (CDS) features; Ring 3: reverse strand ORFs from the primary sequence of Spn1; Ring 4: forward strand ORFs from the primary sequence of Spn1; Ring 5: Contigs; Ring 6 (black): the GC content; Ring 7 (green and purple): the GC skew.

**Fig 2:**
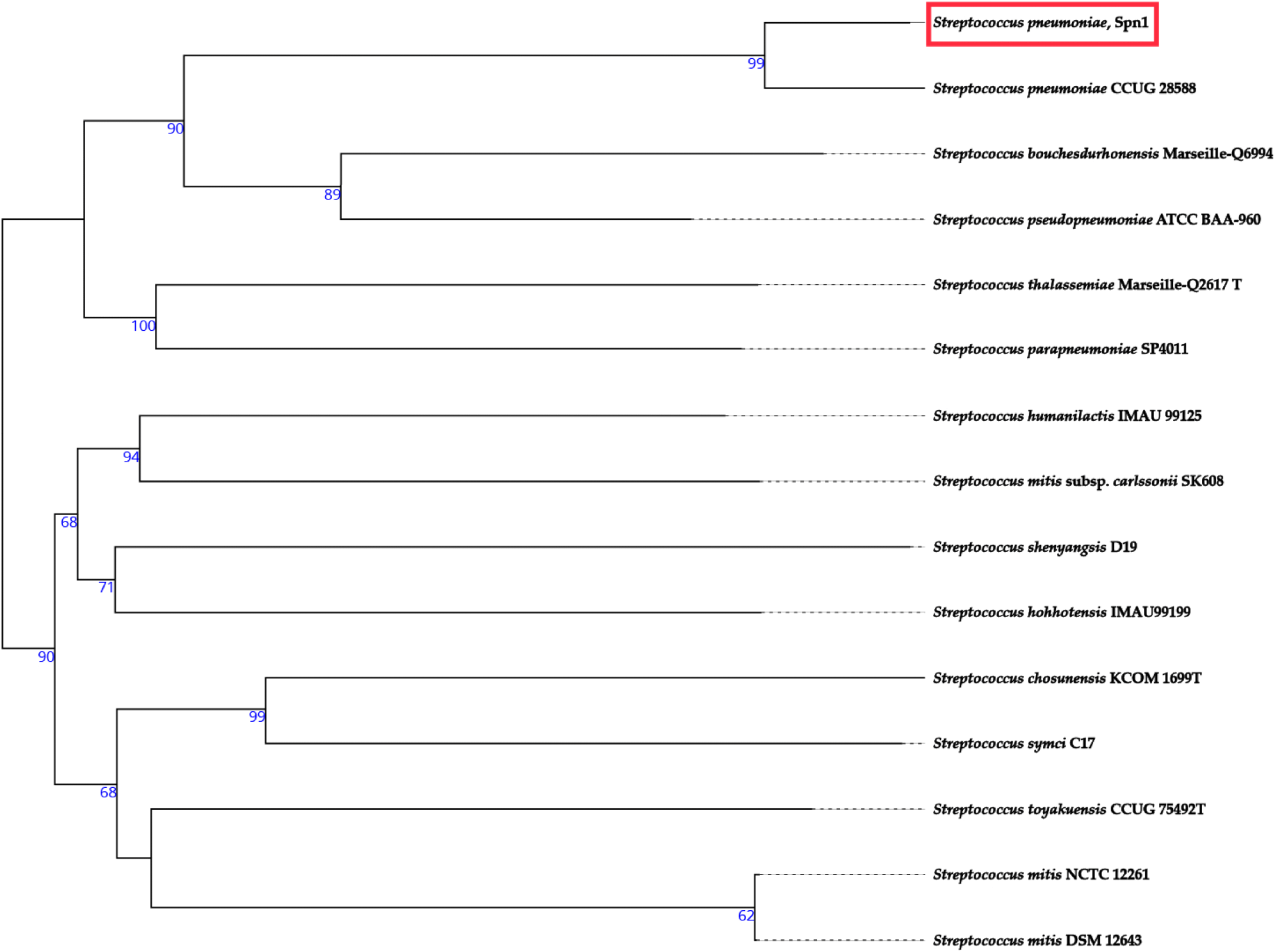
Phylogenomic tree of the *Streptococcus pneumoniae*, Spn1. The tree is based on the GBDP phylogenetic analyses retrieved and modified based on the Type (Strain) Genome Server (TYGS). Tree inferred with FastME 2.1.6.1 from GBDP distances calculated from genome sequences. The branch lengths are scaled in terms of the GBDP distance formula d5. The numbers above the branches are GBDP pseudo-bootstrap support values >60% from 100 replications, with an average branch support of 71.9%. The tree was rooted at the midpoint and visualized with PhyD3.

The genome of *S. pneumoniae* strain Spn1, a clinical isolate received as a kind gift from Dr. Samir K. Saha (Dhaka Shishu Hospital, Dhaka, Bangladesh), was sequenced in this research. The serotype of Spn1 is 19A. The analysis identified the isolate as *S. pneumoniae* with the allelic profile *aroE*_15, *ddl*_14, *gdh*_11, *gki*_9, *recP*_10, *spi*_6, and a potential new *xpt* allele showing 99.8% identity to *xpt_1*. This allelic combination did not correspond to any known sequence type (ST) in the PubMLST database and was therefore designated as an unassigned (unknown) ST, most closely related to ST9953, ST8789, and ST12899. The genome sequence length of the Spn1 strain is 2097441 bp (2.09 Mb). The number of total genes is 2071, and the GC content is 39.52%. The map of the genome, phylogenetic tree, and cluster of orthologous groups are shown in Fig 1, Fig 2, and S1 Fig, respectively. The genome of Spn1 is comprised of 56 contigs with 2071 genes, and it is closely related to *S. pneumoniae* strain, CCUG 28588 (Fig 2). The Spn1 strain belongs to a novel sequence type because only 4 out of 7 loci match with ST-0267. Spn1 does not belong to the clonal complex of ST-0267, and it is a triple locus variant, indicating distant relatedness but not direct lineage. Two incomplete prophage regions (S2 Fig) and a plasmid (S3 Fig), harboring a hypothetical protein of 1206 bp length with 99.83% identity with the repUS423 plasmid (NCBI accession number CP003594), (S4 Table) was identified. Certain antimicrobial genes are identified in the Spn1 genome, including genes conferring resistance to macrolides (*ermB*, *mefA*, *msrD_2*), tetracyclines (*tetM*), chloramphenicol (*cat-TC*), and fluoroquinolones (*patA*), validated across multiple AMR databases with 100% coverage (S5 Table, S6 Table) and these findings are consistent with the AMR profile of Spn1 (S2 Table). In the Spn1 genome, the percentage of virulence genes is 24% (31/129). Several virulence genes are identified in the genome of the Spn1 strain, including pivotal adherence factors (*pspA, pspC, pavA*), toxins (*ply*), immune evasion proteases (*iga, cppA*), and nutrient uptake systems (*piaA, piuA, psaA*) (Table 1). Using CRISPRCasFinder [47], we identified a Type I CRISPR-Cas system in Spn1, comprising a single CRISPR array (position 22,880–22,964) with one spacer and a dedicated *cas* operon (position 15,076–16,419) encoding two Cas3 proteins. The spacer’s repeat consensus (5’-CTTTTTTTGAAACGTTTCATTTTT-3’) aligns with typical Type I repeats, while the Cas3 proteins confirm functional classification. The spatial separation between the CRISPR array and *cas* genes is consistent with Type I genomic architecture, suggesting a potentially functional system despite low spacer acquisition activity (S7 Table). The genome sequence data revealed that virulence genes are present in the genome of *S. pneumoniae* strain, Spn1.

**Table 1:** Virulence genes in the genome of *S. pneumoniae*, Spn1 strain.

Virulence Factor class	Virulence factors	Related genes
Adherence	Choline-binding proteins	*cbpD, cbpG, lytA, lytB, lytC, pce/cbpE, pspA, pspC/cbpA*
	Fibronectin-binding proteins	*pavA*
	Laminin-binding protein	*lmb*
	Streptococcal lipoprotein rotamase A	*slrA*
	Streptococcal plasmin receptor/GAPDH	*plr/gapA*
	rlrA islet	*rrgA, rrgB, rrgC, srtB, srtC, srtD*
Enzyme	Hyaluronidase	*hysA*
	Neuraminidase A	*nanA*
	Streptococcal enolase	*eno*
Immune evasion	Capsule	Undetermined
Iron uptake	Pneumococcal iron acquisition	*piaA*
	Pneumococcal iron uptake	*piuA*
Manganese uptake	Pneumococcal surface antigen A/ Metal binding protein SloC	*psaA*
Protease	C3-degrading protease	*cppA*
	IgA1 protease	*iga*
	Serine protease	*htrA/degP*
	Trigger factor	*tig/ropA*
	Zinc metalloproteinase	*zmpB*
Toxin	Pneumolysin	*ply*

### *S. pneumoniae* kills *Bombyx* larvae when injected into the hemolymph

**Fig 3:**
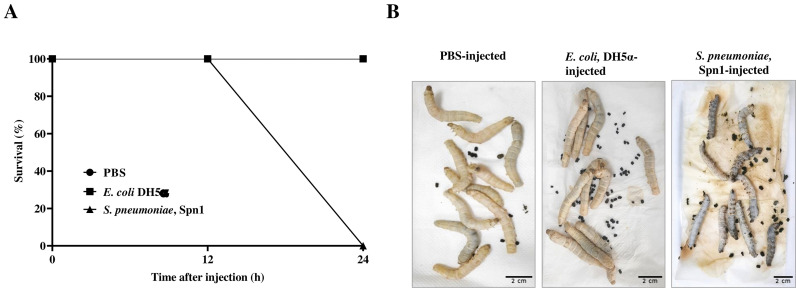
*Streptococcus pneumoniae* strain 1, Spn1 kills *Bombyx mori* larvae. **(A)** Percentage survival of *B. mori* larvae (*n* = 10) 24 h after injection into hemolymph (blood) with 3.0 × 10⁸ CFU of *S. pneumoniae* Spn1 or *E. coli* DH5α suspended in PBS or an equal volume of PBS. The error bar represents the SEM. Data shown are averages of three independent biological experiments. **(B)** Representative images of larvae after 24 h of injection with PBS, *E. coli* DH5α, or Spn1.

**Fig 4:**
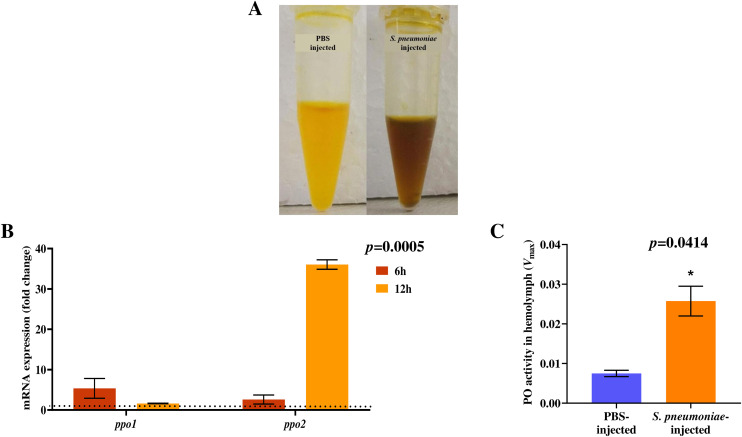
*S. pneumoniae,* Spn1 infection leads to melanization of *Bombyx* larvae. **(A)** melanization (blackening) of the larval hemolymph 12 h post-infection (p.i.). **(B)** RT-qPCR analysis of *ppo1* and *ppo2* gene expression in the larval fat body after 6- and 12 h of infection with 3.0 × 10^8^ CFU of Spn1 through hemolymph. The reference gene used for normalization was *Bmrp49*. The error bars represent the SEM. Data generated from at least three independent biological experiments, each with two technical replicates, and the statistical significance was determined via two-way ANOVA that showed expression was significantly higher at 12h compared to 6h (*p* = 0.0005). **(C)** Increased phenoloxidase (PO) enzyme activity in hemolymph 12 h post-infection (p.i.). The error bars represent the SEM, and the statistical significance was determined via an unpaired t-test, *p < 0.05.

To examine the pathogenicity of *S. pneumoniae* Spn1 strain, we used the *Bombyx* larvae infection model. All the larvae were dead within 24 h of injection of 3.0 × 10^8^ CFU of Spn1 into the hemolymph (Fig 3A), with melanization (blackening) of the cuticle of dead larvae (Fig 3B). No larval death was observed upon injection with 3.0 × 10^8^ CFU of *E. coli* DH5α or PBS (Fig 3A). The lethal dose (LD_50_) of Spn1 for silkworm larvae was determined to be 7.8 × 10^7^ CFU. Collection of hemolymph 12 h post-infection (p.i.) revealed melanization or blackening due to increased melanin formation (Fig 4A). Among the *prophenoloxidase* (*ppo*) genes, *ppo1* and *ppo2* expression were significantly upregulated after 6 h and 12 h p.i., respectively (Fig 4B). The total phenoloxidase (PO) enzyme activity in the hemolymph demonstrated a significant increase 12 h p.i. (Fig 4C). *S. pneumoniae* infects mammals through the lungs, and therefore, we examined the effect of topical application of Spn1 onto spiracles that have openings on the cuticle of the larval body and are connected to tracheal bushes inside. Infection of trachea by Spn1 through topical application onto spiracles of larvae resulted in no death (S4 Fig), but we observed increased melanization of trachea with increased bacterial localization and load (S5 Fig) along with increased tracheal expression of both *ppo1* and *ppo2* genes 6 h and 12 h p.i. (S6 Fig) in comparison to control larvae injected with PBS. To examine whether other *S. pneumoniae* strains can kill *Bombyx* larvae or not, we used three more clinical isolates labeled as Spn2, Spn3, and Spn4. All these strains killed *Bombyx* larvae within 24 h of infection (S7 Fig). The LD_50_ value of the *S. pneumoniae* Spn2, Spn3, and Spn4 strains for *Bombyx* larvae was 1.14 × 10^7^, 3.55 × 10^7^, and 4.22 × 10^7^ CFU, respectively. These results indicate that *Bombyx* larvae can be a useful infection model for studying *S. pneumoniae* pathogenicity.

### Reduced viability of circulating hemocytes of *Bombyx* larvae upon *S. pneumoniae* infection

**Fig 5:**
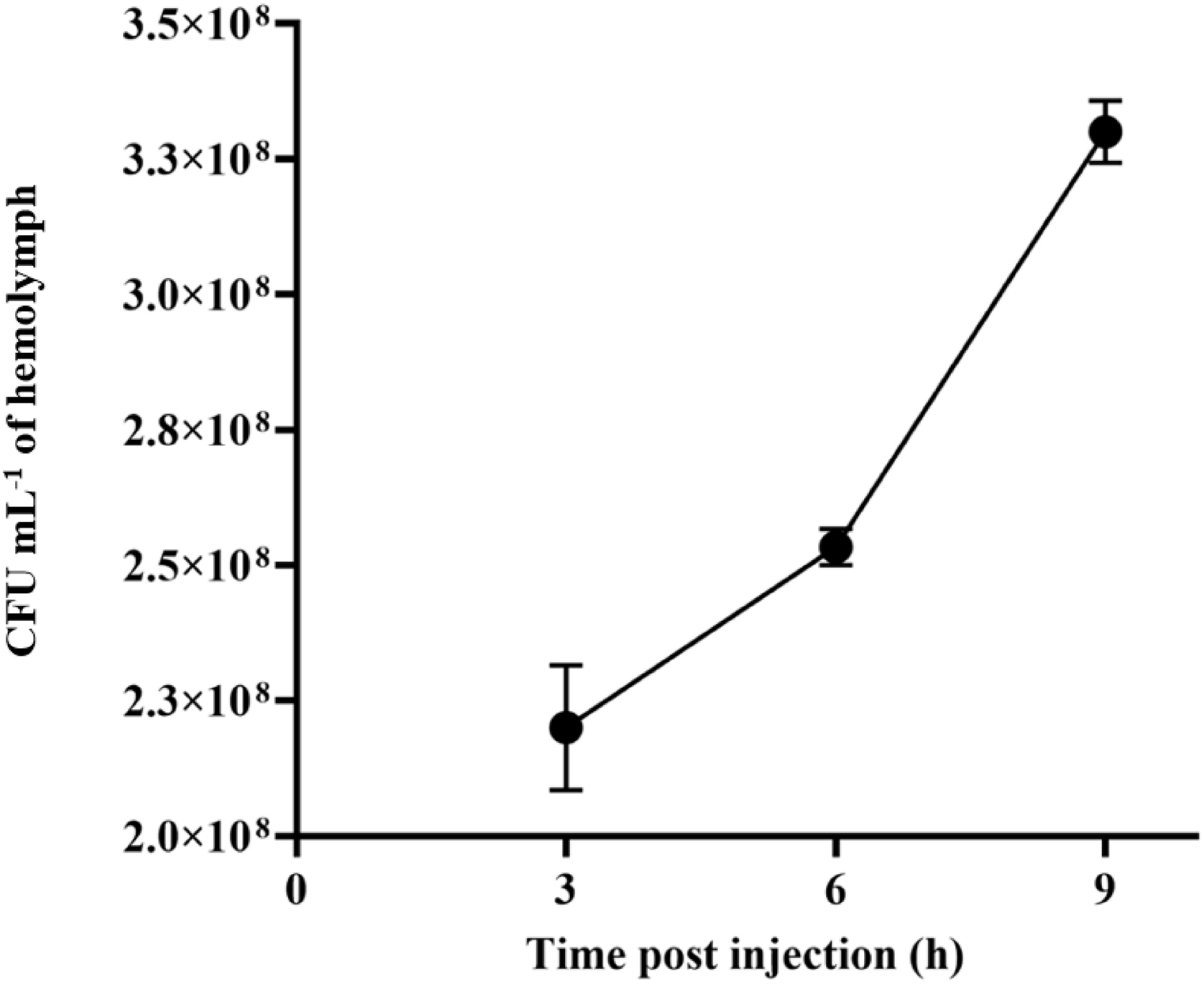
Proliferation of *S. pneumoniae,* Spn1 in *Bombyx* larval hemolymph. Larvae (*n* = 10) were injected with 3.0 × 10^8^ CFU of Spn1 strain, followed by counting of bacterial numbers in the hemolymph by serial dilution and plating. The error bars represent the SEM. Data shown are averages of three independent biological experiments.

**Fig 6:**
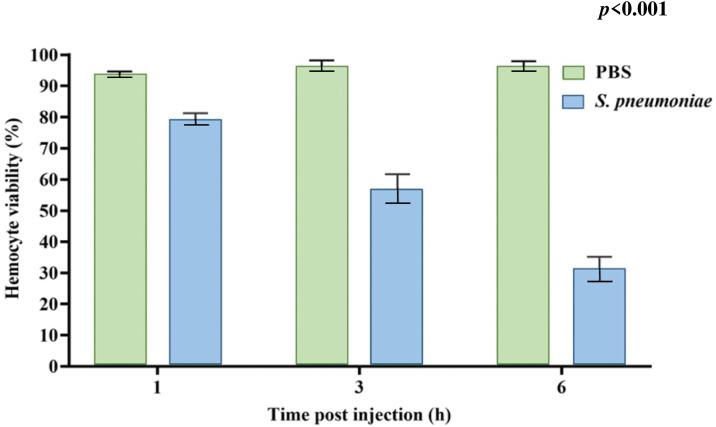
Proliferation of *S. pneumoniae,* Spn1 reduces viability of hemocytes in *Bombyx* larvae. The percentage of viable hemocytes in the larval hemolymph after Spn1 infection (3.0 × 10^8^ CFU) was determined by Trypan Blue staining using hemocytometer. The error bars represent the SEM. Data shown are averages of three independent biological experiments, and the statistical significance was determined via two-way ANOVA. The analysis revealed significant difference between control (PBS) and infected (*S. pneumoniae*) groups (*p* < 0.001), along significant effects of time (*p* < 0.001), supporting that Spn1 infection induces a progressive decline in hemocyte viability with time.

The injection of 3.0 × 10^8^ CFU Spn1 into the larval hemolymph led to a notable rise in bacterial count within 9 h p. i. (Fig 5). Since bacterial infection can kill immune cells like the circulating hemocytes, we examined their viability by Trypan blue staining. The viability of circulating hemocytes was reduced to 30% in Spn1-infected larvae compared to the control larvae (Fig 6). The results indicate that the circulating hemocytes of *Bombyx* larvae can be killed by *S. pneumoniae*.

### Effects of antibiotics on *S. pneumoniae*-induced death of *Bombyx* larvae

**Fig 7:**
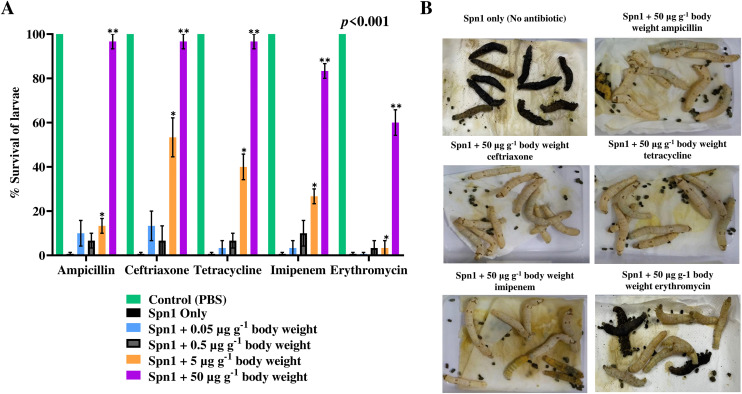
Therapeutic effect of antibiotics on the survival of *Bombyx* larvae injected with *S. pneumoniae,* Spn1. Larvae (*n* = 10) were injected with 3.0 × 10⁸ CFU of Spn1 through an abdominal leg, followed by injection of indicated doses of antibiotics through another abdominal leg. **(A)** Percentage survival of larvae after antibiotic injection 48 h post-infection (p.i.).The error bars represent the SEM. Data are representative of three independent biological experiments. Statistical significance was determined via two-way ANOVA, revealing significant effects of both antibiotic type and dose (*p* < 0.001), with a strong interaction (*p* < 0.001). Pairwise comparisons were analyzed using Tukey’s post hoc test. Increasing antibiotic doses markedly enhanced larval survival after *S. pneumoniae* infection. *Significance levels: *p < 0.05, **p < 0.005*.* **(B)** A dose of 50 µg g^-1^ body weight of all antibiotics except erythromycin showed none or significantly reduced death and melanization 48 h post-infection (p.i.).

According to the Clinical and Laboratory Standards Institute (CLSI) standards [48,49], the *S. pneumoniae* strain, Spn1 used in this study showed resistance to tetracycline, and erythromycin, but sensitivity towards ampicillin, ceftriaxone, and imipenem as determined by both the antimicrobial resistance (AMR) profile (S2 Table) and the minimal inhibitory concentration (MIC) data (Table 2). Next, we examined the antibiotic sensitivity of Spn1 *in vivo* using *Bombyx* larvae. Almost 100% survival was observed for Spn1-infected larvae treated with the highest dose (50 μg g^-1^ body weight) of ampicillin, ceftriaxone, and tetracycline, whereas 80% and 60% survival was observed with imipenem and erythromycin, respectively (Fig 7A), with a significant reduction of melanization (Fig 7B). Spn1 displayed *in vitro* resistance to tetracycline and erythromycin, but the *Bombyx* larvae model showed it was sensitive to these antibiotics *in vivo*. The effective dosage (ED₅₀) values for the five antibiotics tested in the larvae against Spn1 are listed in Table 2. The findings suggest that the sensitivity of *S. pneumoniae* to antibiotics can be assessed using the *Bombyx* larvae infection model.

**Table 2:** MIC of antibiotics against *S. pneumoniae*, Spn1, and ED_50_ of antibiotics in *Bombyx* larvae against *S. pneumoniae.*

Antibiotics	MIC (µg/mL)	ED_50_ (µg/g of larva)
Ampicillin	0.195	3.10
Ceftriaxone	0.390	1.46
Tetracycline	6	0.421
Erythromycin	3.75	7.24
Imipenem	0.0098	3.25

### *S. pneumoniae* upregulates homologs of mammalian *TLR2*, *TLR4* and AMP genes in *Bombyx* larvae

**Fig 8:**
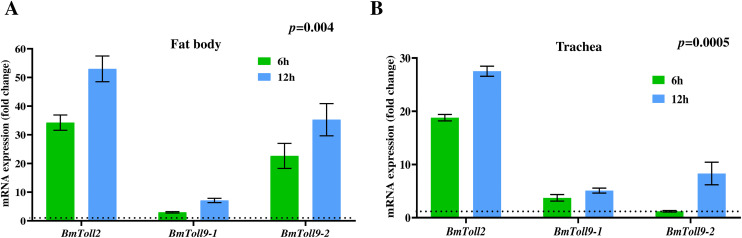
*S. pneumoniae* upregulates *BmToll2*, *BmToll9*−1, and *BmToll9*−2 in fat body and trachea of *Bombyx* larvae. RT-qPCR analysis of *BmToll2*, *BmToll9*−1, and *BmToll9*−2 in fat body (A) and trachea (B) isolated from larvae 6 h and 12 h after injection of 3.0x10^8^ CFU of Spn1 through the hemolymph. The reference gene used for normalization was *Bmrp49*. The error bars represent the SEM. Data are generated from three independent biological experiments, each with two technical replicates, and the statistical significance was determined via two-way ANOVA. The dashed line represents an expression level of 1.00. **(A)** For fat body, the overall expression was significantly higher at 12 h compared to 6 h (*p* = 0.004). **(B)** For trachea, the overall expression was significantly higher at 12 h compared to 6 h (*p* = 0.0005).

The Toll-like receptor (TLR) family members, TLR2 and TLR4, participate in the innate immune response against *S. pneumoniae* in mammals by playing critical roles in recognizing and responding to infections by this important pathogen [30,32]. The *BmToll2* and *BmToll9* genes (*BmToll9−1* and *BmToll9−2* isoforms) are *Bombyx* homologs of mammalian *TLR2* and *TLR4* [34,50]. This study demonstrates that injection of the *S. pneumoniae* strain, Spn1, into the hemolymph of *Bombyx* larvae resulted in the increased expression of *BmToll2* by more than 35- and 50-fold in the fat body and 6 h and 12 h p.i., respectively, in comparison to the uninfected larvae (Fig 8A). For *BmToll9−1*, no significant upregulation was observed 6 h p.i., and more than 5-fold increased expression was observed after 12 h p.i. (Fig 8A). *BmToll9−2* expression was induced in the fat body by ~20-fold after 6 h, and more than 30-fold after 12 h p.i. (Fig 8A). In the trachea, after hemolymph infection by Spn1, *BmToll2* exhibited upregulation of approximately 19-fold and 28-fold at 6 h and 12 h, respectively. In contrast, *BmToll9−1* expression increased by approximately 3-fold and 5-fold at 6 h and 12 h of infection, respectively (Fig 8B). No upregulation of *BmToll9−2* was observed in the trachea after 6 h of infection with Spn1, but the expression was increased by ~10-fold after 12 h (Fig 8B). Topical application of Spn1 onto the spiracles significantly induced the expression of *BmToll2*, *BmToll9−1*, and *BmToll9−2* after 12 h of infection in both the fat body and trachea (S8 Fig).

**Fig 9:**
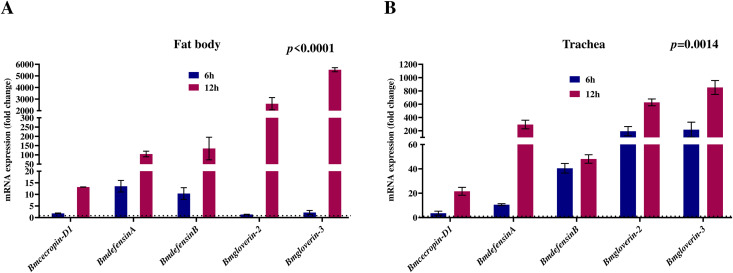
*S. pneumoniae* upregulates antimicrobial peptide (AMP) genes in fat body and trachea of *Bombyx* larvae. RT-qPCR analysis of different AMP genes in the fat body (A) and trachea (B) isolated from larvae 6 h and 12 h after injection with 3.0 × 10⁸ CFU of Spn1. The reference gene used for normalization was *Bmrp49*. The error bars represent the SEM. Data are generated from at least three independent biological experiments, each with two technical replicates, and the statistical significance was determined via two-way ANOVA. The dashed line represents an expression level of 1.00. **(A)** For fat body, two-way ANOVA revealed significant effects of time (*p* < 0.0001), indicating that with time, expression of genes significantly increased. **(B)** For trachea, two-way ANOVA revealed significant effects of time (*p* = 0.0014), indicating with time, expression of genes significantly increased.

Next, we examined whether the activation of *BmToll2*, *BmToll9−1*, and *BmToll9−2* in *Bombyx* larval fat body and trachea upon infection by *S. pneumoniae* can upregulate expression of the antimicrobial peptide (AMP) genes, including *Bombyx defensin* genes whose homologs in mammals are known to be upregulated after *S. pneumoniae* infection [51,52]. In the larval fat body after Spn1 infection through hemolymph, the expressions of *BmdefensinA* and *BmdefensinB* were induced by ~10- and ~100-fold after 6 h and 12 h, respectively, whereas the expressions of *Bmcecropin-D1*, *Bmgloverin-2*, and *Bmgloverin-3* were induced by ~10-, ~ 2000-, and ~5000-fold after 12 h of infection (Fig 9A). A similar expression pattern of the AMP genes was observed in the trachea after Spn1 infection through hemolymph (Fig 9B). These results indicate that the *S. pneumoniae*-induced upregulation of the homologs of mammalian *TLR2* and *TLR4* is evolutionarily conserved in insects like *B. mori*.

## Discussion

Among the streptococci, *S. pyogenes*, a Group A streptococci, is a serious pathogen of humans, and the silkworm infection model has been used to study its pathogenicity [53], but no such studies have been carried out for *S. pneumonia*e, a human respiratory pathogen. This study applies the *Bombyx* larvae infection model to investigate the pathogenicity of *S. pneumoniae*,and we have shown that, similar to mammals, the *Toll* signaling pathway components, *BmToll2* and *BmToll9* isoforms, homologs of mammalian TLR2 and TLR4, respectively, are upregulated in the *Bombyx* larvae upon *S. pneumoniae* infection.

In this study, the clinical isolate of *S. pneumoniae*, Spn1, that we used belongs to serotype 19A, that has been reported to cause invasive pneumococcal disease, pneumonia, otitis media, and hemolytic uremic syndrome in various countries [54]. For a successful infection of hosts, *S. pneumoniae* expresses a battery of virulence genes to compromise the host immunity, and the analysis of the whole genome of the Spn1 strain used in this study revealed several such genes (Table 1). The *ply* gene that encodes a pore-forming toxin of *S. pneumoniae*, pneumolysin, is detected in the Spn1 genome. Multiple genes encoding for choline-binding proteins, including *cbpG* and *cbpD* are also present that are important for colonization and invasion. The presence of genes encoding enzymes like hyaluronidase and enolase facilitates the spread of Spn1 inside the host. The genes, *piaA* and *piuA*, responsible for the sequestering of iron from the host and the manganese-uptake gene, *psaA*, needed for antioxidant defense are also detected (Table 1). The genes encoding for proteases, including *htrA*, *cppA*, and *zmpB*, that are crucial for host protein degradation, evasion of the host immune system, and essential for virulence, respectively, are also present in the Spn1 genome. The presence of all these virulence genes in the genome of Spn1 confirmed that it is a pathogenic strain of *S. pneumoniae*.

In this study, we showed that four different clinical isolates of *S. pneumoniae* can kill silkworm larvae within 24 h (Fig 3, S7 Fig). Kaito et al. reported that a clinical isolate of *S. pyogenes* required more than 96 h to kill larvae with a dose similar to those used in this study for *S. pneumoniae* [53]. Experiments conducted at the same time with the same silkworm strain with different strains of *S. pyogenes* and *S. pneumoniae* may reveal which strain is more pathogenic. Insect PO activity is increased upon bacterial infection [55]. We observed blackening (melanization) of both hemolymph and cuticle of larvae upon *S. pneumoniae* infection with an increase of PO activity (Fig 4). The extent of melanization of the cuticle of the dead silkworm larvae after *S. pneumoniae* infection is visibly much lower (Fig 3, S7 Fig) compared to other pathogenic bacterial infections that we reported previously [13–15]. Reduced melanization after *S. pneumoniae* infection was also reported in *D. melanogaster* [38]. We also observed a drop of bacterial load in the hemolymph 3 h post-infection (p.i.) (Fig 5), which could be due to the attachment of bacteria to internal host tissues at the onset of infection, as has been observed with some other pathogenic bacteria in *Bombyx* larvae [13,16,56]. In mammals, *S. pneumoniae* infection can kill host cells like red blood cells by producing hydrogen peroxide and toxins [57], and likewise, we observed killing of almost 70% of hemocytes in *Bombyx* larvae within 3 h of *S. pneumoniae* infection in our study (Fig 6). It would be worthwhile to examine the mechanism of the death of the larval hemocytes by *S. pneumoniae*.

The initial investigation on the therapeutic efficacy of antibiotics against human pathogenic bacteria in silkworm larvae was conducted by Hamamoto et al. [12]. No study has been carried out to study the therapeutic effect of antibiotics against any *Streptococcus species* or the host immune response against it in the *Bombyx* larvae infection model. Although the three antibiotics (ampicillin, ceftriaxone, imipenem) against which the Spn1 strain showed sensitivity *in vitro* also showed therapeutic effect (80–90% survival) against the bacterium *in vivo* in the silkworm larvae infection model (Table 2, Fig 7), tetracycline and erythromycin were exceptions. The whole genome sequence analysis revealed the presence of both tetracycline- and erythromycin-resistant genes (S5 Table, S6 Table) in the Spn1 genome. Against tetracycline and erythromycin, the Spn1 strain showed resistance *in vitro* (S2 Table, Table 2), however, we observed ~100% and ~60% therapeutic effect against these two antibiotics in Spn1-infected silkworm larvae (Fig 7A). At therapeutic doses, antibiotics reduced the blackening of larvae (Fig 7B), supporting the notion that the reduction of bacterial load by antibiotics led to a decrease in melanization. Our group recently encountered a similar mismatch between in vitro and in vivo antibiotic sensitivity findings using *E. coli* O157:H7, *K. pneumoniae*, *N. circulans*, and *K. aerogenes* [13–16]. Such a disparity between in vitro and in vivo antibiotic susceptibility data is widely considered a common occurrence [58]. These results underscore the necessity of employing infection models to precisely gauge the antibiotic susceptibility of bacteria that are pathogenic to humans. In this context, *Bombyx* larvae may serve as a fitting infection model for the initial screening of known antibiotics or biological extracts that are effective against multidrug-resistant (MDR) or extensively drug-resistant (XDR) strains of *S. pneumoniae* or other human pathogens before validating the results in a mouse model. We have recently shown, for instance, that aqueous extracts from hog plum or Indian gooseberry are capable of rescuing *Bombyx* larvae infected with *K. aerogenes* [16].

Antimicrobial peptides (AMPs) contain evolutionarily conserved components of innate immunity in multicellular organisms [59,60]. In insects, the primary sites for the AMP production in response to infection are the fat body tissues that are equivalent to mammalian liver and adipose tissue [61]. Among the AMPs, defensins and gloverins are generally most effective against Gram-positive and Gram-negative bacteria, respectively [62]. *Streptococcus pneumoniae* enhances the expression of *β defensin-2* and *β defensin-3* in human lung epithelial cells, with *β defensin-2* levels increasing after TLR2 activation in human tracheobronchial epithelial cells [51,52]. Our observations that *S. pneumoniae* significantly upregulates *BmdefensinA* and *BmdefensinB* genes in *Bombyx* fat body (Fig 9) are consistent with these reports. Vertebrate β defensins are structurally more similar to insect defensins than α defensins and θ defensins, indicating that defensins are evolutionarily conserved [62]. *B. mori* has eleven *Toll* genes, and overexpression of *BmToll2* induces expression of *defensin* genes in *Bombyx* cells *in vitro* [50,63]. Our observations that *BmToll2* expression is increased upon *S. pneumoniae* infection in *Bombyx* fat body (Fig 8) are consistent with these reports, and it is plausible that *S. pneumoniae*-induced expression of *defensin* genes is *BmToll2* dependent. It would be worthwhile to know whether *BmToll2* knockdown or knockout can affect the upregulation of *defensin* genes after *S. pneumoniae* infection. The *Drosophila Toll9* can activate the promoters of *defensin* and other AMP genes when overexpressed in cultured cells [64], however, no antimicrobial response defect was observed in *Drosophila Toll9* mutant [65]. *BmToll9* shares structural features with mammalian TLR4 [34]. Mammalian TLR4 is involved in defense against *S. pneumoniae* [66]. We found that *BmToll9* is significantly upregulated in the *Bombyx* fat body in response to the injection of *S. pneumoniae* into the hemolymph (Fig 8). *S. aureus*, a Gram-positive bacterium, also upregulates *BmToll9* in the fat body, whereas no such upregulation was observed with *E. coli*, a Gram-negative bacterium [67]. The function of *BmToll9* in response to *S*. *pneumoniae* infection warrants further studies.

The major route of transmission of pneumococcus is through direct respiratory droplets in humans. Silkworms breathe through trachea through spiracle, the openings of the tracheal system on the integument of the insect [68]. We found that the topical application of *S. pneumoniae* onto spiracles resulted in the infection of both trachea and hemolymph concomitant with an increased darkening (melanization) of trachea (S5 Fig), although none of the larvae died, probably due to the low dose of topical application of bacteria. In mice, intranasal inoculation of *S. pneumoniae* activates the coagulation pathway in the lungs in a TLR2-dependent manner to prevent the spread of the bacteria [69]. There are significant similarities in the cellular mechanisms between blood coagulation and insect hemolymph coagulation [70]. Interestingly, in this study, we found that topical application of *S. pneumoniae* onto the spiracles resulted not only in the increased melanization of the trachea, indicating localization of hemocytes onto the trachea (S5 Fig) but also upregulation of *BmToll2*, and *BmToll9−2* isoform (S8 Fig). Establishment of *BmToll2* and *BmToll9* mutant silkworms would conclusively reveal their importance in the upregulation of *Bombyx defensins* and *gloverins* genes during pneumococcal infection.

In conclusion, our study shows that the larvae of *Bombyx mori* can be used as an infection model to screen for antibiotics or unknown biological extracts with antibacterial compound(s) that are effective against *S. pneumoniae* clinical isolates. We showed that, similar to vertebrates, *S. pneumoniae* upregulates *BmToll2*, *BmToll9*, and *Bmdefensins* in *Bombyx* larvae, further strengthening the notion that the model is suitable for studying the pathogenicity of this important pathogen.

## Supporting information

S1 Fig Cluster of orthologous groups (COG) of *Streptococcus pneumoniae* strain 1, Spn1. The horizontal axis displays Clusters of Orthologous Gene function type, and the vertical axis is the number of annotated genes.
(TIF)

S2 Fig Schematic representation of the predicted prophages in *Streptococcus pneumoniae*, Spn1 genome identified using PHASTER. 2 prophage regions have been identified, of which 0 regions are intact, 2 regions are incomplete, and 0 regions are questionable. (https://phaster.ca/batches/BB_09a87038f7). In the Spn1 strain, 2 incomplete prophage regions have been identified. (A) One prophage with 14 CDS extending from 90174 bp to 106620 bp (16.4Kb). This prophage consisted of hypothetical proteins, transposase, integrase, tail protein, and phage-like proteins. (B) Another prophage with 9 CDS extending from 35978 bp to 45261 bp (9.2Kb). This prophage consisted of hypothetical proteins and phage-like proteins.
(TIF)

S3 Fig Circular Map showing the plasmid (repUS43) of *Streptococcus pneumoniae*, Spn1. The contents of the feature rings (starting with the outermost ring) are as follows: Ring 1: Forward strand of coding sequence (CDS) features; Ring 2: Contigs; Ring 3 (red): the GC content; Ring 4 (green and purple): the GC skew; Ring 5: forward strand ORFs from the plasmid (repUS43) sequence.
(TIF)

S4 Fig Topical application of *S. pneumoniae,* Spn1 suspension onto spiracles does not kill *Bombyx* larvae. Percentage survival of *Bombyx* larvae (*n* = 10) 24 h after topical application of 3.0 × 10⁸ CFU or 3.0 × 10⁹ CFU of Spn1 suspended in PBS or an equal volume of PBS. The error bars represent the SEM. Data generated from three independent biological experiments.
(TIF)

S5 Fig Topical application of *S. pneumoniae,* Spn1 onto spiracles causes melanization (increased darkening) of *Bombyx* tracheal bushes. (A) Darkening of tracheae in larvae after 12 h in response to infection through spiracles. Proliferation of bacteria in hemolymph (B) and trachea (B’) 6 and 12 h post-infection (p.i.) after topical application of bacterial suspension 3.0 × 10⁸ CFU or 3.0 × 10⁹ CFU onto spiracles. The error bars represent the SEM. Data are generated from at least three independent biological experiments, and the statistical significance was determined via two-way ANOVA. For *S. pneumoniae* proliferation in hemolymph (B) and trachea (B’), two-way ANOVA revealed significant effects of time (*p* < 0.0001), indicating with time, CFU has significantly increased. The statistical analysis also revealed significant presence of Spn1 in infected larvae as opposed to control (PBS) group (*p* < 0.0001).
(TIF)

S6 Fig Upregulation of *ppo1* and *ppo2* in trachea isolated from larvae 6 h and 12 h after injection through hemolymph with 3.0 × 10⁸ CFU of *S. pneumoniae*, Spn1. The reference gene used for normalization was *Bmrp49.* The error bars represent the SEM. Data generated from at least three independent biological experiments, each with two technical replicates, and the statistical significance was determined via two-way ANOVA that showed expression was significantly higher at 12h compared to 6h (*p* = 0.0228). The dashed line represents an expression level of 1.00.
(TIF)

S7 Fig Three clinical isolates of *S. pneumoniae*, Spn2, Spn3, and Spn4 kill silkworm larvae. (A) Percentage survival of *Bombyx* larvae (*n* = 10) 24 h post-infection (p.i.) with Spn2 (5.1 × 10⁸ CFU), Spn3 (6.60 × 10⁸ CFU), Spn4 (4.5 × 10⁸ CFU), *E. coli* DH5α (6.60 × 10⁸ CFU), and PBS. The error bars represent the SEM. Data generated from three independent biological experiments. (B) Images of fifth instar larvae 24 h after injection with PBS, *E. coli* DH5α, Spn2, Spn3, or Spn4.
(TIF)

S8 Fig Effect of topical application of *S. pneumoniae* onto spiracles on the expression of *BmToll2*, *BmToll9−1*, and *BmToll9−2* in *Bombyx* larvae. RT-qPCR analysis of *BmToll2, BmToll9−1*, and *BmToll9−2* in the fat body (A) and trachea (B) isolated from larvae 6 h and 12 h after topical application of 3.0x10^8^ CFU of Spn1. The reference gene used for normalization was *Bmrp49.* The error bars represent the SEM. Data generated from at least three independent biological experiments, each with two technical replicates, and the statistical significance was determined via two-way ANOVA. The dashed line represents an expression level of 1.00. (A) For fat body, the overall expression was significantly higher at 12 h compared to 6 h (*p* < 0.0001). (B) For trachea, the overall expression was significantly higher at 12 h compared to 6 h (*p* < 0.0001).
(TIF)

S1 Table Biochemical tests of the *S. pneumoniae*, Spn1 strain, used in this study. (DOCX)

S2 Table Antimicrobial resistance (AMR) profile of *S. pneumoniae,* Spn1 strain used in this study. (DOCX)

S3 Table Primers used for RT-qPCR in this study. (DOCX)

S4 Table RepUS423 plasmid identified in the *S. pneumoniae*, Spn1 strain used in this study. (DOCX)

S5 Table Annotation of Spn1 genome data using the Rapid Annotation using Subsystem Technology (RAST) server. (XLS)

S6 Table Summary of the antimicrobial resistance genes present in the genome of *Streptococcus pneumoniae*, Spn1 strain used in this study identified by four different databases. (DOCX)

S7 Table The CRISPR/Cas system of *S. pneumoniae*, Spn1 strain used in this study. (DOCX)

## References

[1] World Health Organization. Pneumonia. https://www.who.int/news-room/fact-sheets/detail/pneumonia 2023 October 1.

[2] Kadioglu A, Taylor S, Iannelli F, Pozzi G, Mitchell TJ, Andrew PW. Upper and lower respiratory tract infection by Streptococcus pneumoniae is affected by pneumolysin deficiency and differences in capsule type. Infect Immun. 2002;70(6):2886–90. doi: 10.1128/IAI.70.6.2886-2890.2002 12010976 PMC128015

[3] Bergenfelz C, Hakansson AP. Streptococcus pneumoniae Otitis Media Pathogenesis and How It Informs Our Understanding of Vaccine Strategies. Curr Otorhinolaryngol Rep. 2017;5(2):115–24. doi: 10.1007/s40136-017-0152-6 28616365 PMC5446555

[4] Saha SK, Rikitomi N, Ruhulamin M, Masaki H, Hanif M, Islam M, et al. Antimicrobial resistance and serotype distribution of Streptococcus pneumoniae strains causing childhood infections in Bangladesh, 1993 to 1997. J Clin Microbiol. 1999;37(3):798–800. doi: 10.1128/JCM.37.3.798-800.1999 9986858 PMC84560

[5] Megged O. Characteristics of Streptococcus pyogenes Versus Streptococcus pneumoniae Pleural Empyema and Pneumonia With Pleural Effusion in Children. Pediatr Infect Dis J. 2020;39(9):799–802. doi: 10.1097/INF.0000000000002699 32804461

[6] Kim L, McGee L, Tomczyk S, Beall B. Biological and Epidemiological Features of Antibiotic-Resistant Streptococcus pneumoniae in Pre- and Post-Conjugate Vaccine Eras: a United States Perspective. Clin Microbiol Rev. 2016;29(3):525–52. doi: 10.1128/CMR.00058-15 27076637 PMC4861989

[7] Weiser JN, Ferreira DM, Paton JC. Streptococcus pneumoniae: transmission, colonization and invasion. Nat Rev Microbiol. 2018;16(6):355–67. doi: 10.1038/s41579-018-0001-8 29599457 PMC5949087

[8] Ahmed I, Rabbi MB, Sultana S. Antibiotic resistance in Bangladesh: A systematic review. Int J Infect Dis. 2019;80:54–61. doi: 10.1016/j.ijid.2018.12.017 30634043

[9] Van Boeckel TP, Pires J, Silvester R, Zhao C, Song J, Criscuolo NG, et al. Global trends in antimicrobial resistance in animals in low- and middle-income countries. Science. 2019;365(6459):eaaw1944. doi: 10.1126/science.aaw1944 31604207

[10] Panthee S, Paudel A, Hamamoto H, Sekimizu K. Advantages of the Silkworm As an Animal Model for Developing Novel Antimicrobial Agents. Front Microbiol. 2017;8:373. doi: 10.3389/fmicb.2017.00373 28326075 PMC5339274

[11] Hossain MS, Hamamoto H, Matsumoto Y, Razanajatovo IM, Larranaga J, Kaito C, et al. Use of silkworm larvae to study pathogenic bacterial toxins. J Biochem. 2006;140(3):439–44. doi: 10.1093/jb/mvj171 16891331

[12] Hamamoto H, Kurokawa K, Kaito C, Kamura K, Manitra Razanajatovo I, Kusuhara H, et al. Quantitative evaluation of the therapeutic effects of antibiotics using silkworms infected with human pathogenic microorganisms. Antimicrob Agents Chemother. 2004;48(3):774–9. doi: 10.1128/AAC.48.3.774-779.2004 14982763 PMC353159

[13] Ahad II, Hossain MM, Uddin MA, Bari ML, Hossain MS. Therapeutic Effect of Antibiotics Against Escherichia coli O157:H7 in Silk Moth Larvae Animal Model. Curr Microbiol. 2020;77(9):2172–80. doi: 10.1007/s00284-020-02023-1 32417963

[14] Tuba T, Chowdhury FR, Hossain T, Farzana M, Ahad II, Hossain MM, et al. Klebsiella pneumoniae pathogenicity in silk moth larvae infection model. FEMS Microbiol Lett. 2022;368(21–24):fnab159. doi: 10.1093/femsle/fnab159 34931660

[15] Hossain MI, Saleh NUA, Numan A, Hossain MM, Uddin MA, Hossain MS. <i>Bombyx mori</i> as a model for <i>Niallia circulans</i> pathogenicity. DD&T. 2023;17(1):18–25. doi: 10.5582/ddt.2022.01112 36843035

[16] Kabir T, Hossain MI, Jepu TA, Sarker M, Saleh NUA, Monir H, et al. Genome characterization, pathogenicity, and evaluation of therapeutics of Klebsiella aerogenes in Bombyx larvae infection model. BMC Microbiol. 2025;25(1):209. doi: 10.1186/s12866-025-03942-4 40221642 PMC11992878

[17] Matsumoto Y, Sekimizu K. Silkworm as an experimental animal for research on fungal infections. Microbiology and Immunology. 2019;63(2):41–50. doi: 10.1111/1348-0421.12668 30666711 PMC6594098

[18] Tabuchi F, Mikami K, Miyauchi M, Sekimizu K, Miyashita A. Discovery of new AMR drugs targeting modulators of antimicrobial activity using in vivo silkworm screening systems. J Antibiot (Tokyo). 2025;78(2):69–77. doi: 10.1038/s41429-024-00788-2 39543333 PMC11769840

[19] Hamamoto H, Horie R, Sekimizu K. Pharmacokinetics of anti-infectious reagents in silkworms. Sci Rep. 2019;9(1):9451. doi: 10.1038/s41598-019-46013-1 31263251 PMC6602958

[20] Buchmann K. Evolution of Innate Immunity: Clues from Invertebrates via Fish to Mammals. Front Immunol. 2014;5:459. doi: 10.3389/fimmu.2014.00459 25295041 PMC4172062

[21] Müller U, Vogel P, Alber G, Schaub GA. The innate immune system of mammals and insects. Contrib Microbiol. 2008;15:21–44. doi: 10.1159/000135684 18511854

[22] Browne N, Heelan M, Kavanagh K. An analysis of the structural and functional similarities of insect hemocytes and mammalian phagocytes. Virulence. 2013;4(7):597–603. doi: 10.4161/viru.25906 23921374 PMC3906293

[23] Sheehan G, Garvey A, Croke M, Kavanagh K. Innate humoral immune defences in mammals and insects: The same, with differences ?. Virulence. 2018;9(1):1625–39. doi: 10.1080/21505594.2018.1526531 30257608 PMC7000196

[24] Anderson KV, Jürgens G, Nüsslein-Volhard C. Establishment of dorsal-ventral polarity in the Drosophila embryo: genetic studies on the role of the Toll gene product. Cell. 1985;42(3):779–89. doi: 10.1016/0092-8674(85)90274-0 3931918

[25] Hoffmann JA, Reichhart J-M. Drosophila innate immunity: an evolutionary perspective. Nat Immunol. 2002;3(2):121–6. doi: 10.1038/ni0202-121 11812988

[26] Takeda K. Evolution and integration of innate immune recognition systems: the Toll-like receptors. J Endotoxin Res. 2005;11(1):51–5. doi: 10.1179/096805105225006687 15826379

[27] Nie L, Cai S-Y, Shao J-Z, Chen J. Toll-Like Receptors, Associated Biological Roles, and Signaling Networks in Non-Mammals. Front Immunol. 2018;9:1523. doi: 10.3389/fimmu.2018.01523 30034391 PMC6043800

[28] Alexopoulou L, Irla M. Toll-like receptors (TLRs) in the trained immunity era. Elife. 2025;14:e106443. doi: 10.7554/eLife.106443 40891672 PMC12404611

[29] Aderem A, Ulevitch RJ. Toll-like receptors in the induction of the innate immune response. Nature. 2000;406(6797):782–7. doi: 10.1038/35021228 10963608

[30] Koedel U, Angele B, Rupprecht T, Wagner H, Roggenkamp A, Pfister H-W, et al. Toll-like receptor 2 participates in mediation of immune response in experimental pneumococcal meningitis. J Immunol. 2003;170(1):438–44. doi: 10.4049/jimmunol.170.1.438 12496429

[31] Thorburn AN, Tseng H-Y, Donovan C, Hansbro NG, Jarnicki AG, Foster PS, et al. TLR2, TLR4 AND MyD88 Mediate Allergic Airway Disease (AAD) and Streptococcus pneumoniae-Induced Suppression of AAD. PLoS One. 2016;11(6):e0156402. doi: 10.1371/journal.pone.0156402 27309732 PMC4911048

[32] Sánchez-Tarjuelo R, Cortegano I, Manosalva J, Rodríguez M, Ruíz C, Alía M, et al. The TLR4-MyD88 Signaling Axis Regulates Lung Monocyte Differentiation Pathways in Response to Streptococcus pneumoniae. Front Immunol. 2020;11:2120. doi: 10.3389/fimmu.2020.02120 33042124 PMC7525032

[33] Tanaka H, Ishibashi J, Fujita K, Nakajima Y, Sagisaka A, Tomimoto K, et al. A genome-wide analysis of genes and gene families involved in innate immunity of Bombyx mori. Insect Biochem Mol Biol. 2008;38(12):1087–110. doi: 10.1016/j.ibmb.2008.09.001 18835443

[34] Zhang R, Li X, Zhang J, Li Y, Wang Y, Song Y, et al. Toll9 from Bombyx mori functions as a pattern recognition receptor that shares features with Toll-like receptor 4 from mammals. Proc Natl Acad Sci U S A. 2021;118(19):e2103021118. doi: 10.1073/pnas.2103021118 33963082 PMC8126858

[35] Muhammad A, Sun C, Shao Y. The humoral immune response of the lepidopteran model insect, silkworm Bombyx mori L., to microbial pathogens. Curr Res Insect Sci. 2024;6:100097. doi: 10.1016/j.cris.2024.100097 39364346 PMC11447326

[36] Liu J, Chen W, Situ J, Li J, Chen J, Lai M, et al. BmToll9-1 Is a Positive Regulator of the Immune Response in the Silkworm Bombyx mori. Insects. 2024;15(9):643. doi: 10.3390/insects15090643 39336611 PMC11432072

[37] Liu J, Chen W, Chen S, Li S, Swevers L. Similarly to BmToll9-1, BmToll9-2 Is a Positive Regulator of the Humoral Immune Response in the Silkworm, Bombyx mori. Insects. 2024;15(12):1005. doi: 10.3390/insects15121005 39769607 PMC11678180

[38] Pham LN, Dionne MS, Shirasu-Hiza M, Schneider DS. A specific primed immune response in Drosophila is dependent on phagocytes. PLoS Pathog. 2007;3(3):e26. doi: 10.1371/journal.ppat.0030026 17352533 PMC1817657

[39] Roth A, Reichmann P, Hakenbeck R. The capsule of Streptococcus pneumoniae contributes to virulence in the insect model Manduca sexta. J Mol Microbiol Biotechnol. 2012;22(5):326–34. doi: 10.1159/000345327 23221622

[40] Cools F, Torfs E, Aizawa J, Vanhoutte B, Maes L, Caljon G, et al. Optimization and Characterization of a Galleria mellonella Larval Infection Model for Virulence Studies and the Evaluation of Therapeutics Against Streptococcus pneumoniae. Front Microbiol. 2019;10:311. doi: 10.3389/fmicb.2019.00311 30846978 PMC6394149

[41] Carattoli A, Zankari E, García-Fernández A, Voldby Larsen M, Lund O, Villa L, et al. In silico detection and typing of plasmids using PlasmidFinder and plasmid multilocus sequence typing. Antimicrob Agents Chemother. 2014;58(7):3895–903. doi: 10.1128/AAC.02412-14 24777092 PMC4068535

[42] Chen L, Yang J, Yu J, Yao Z, Sun L, Shen Y, et al. VFDB: a reference database for bacterial virulence factors. Nucleic Acids Res. 2005;33(Database issue):D325-8. doi: 10.1093/nar/gki008 15608208 PMC539962

[43] Alcock BP, Huynh W, Chalil R, Smith KW, Raphenya AR, Wlodarski MA, et al. CARD 2023: expanded curation, support for machine learning, and resistome prediction at the Comprehensive Antibiotic Resistance Database. Nucleic Acids Res. 2023;51(D1):D690–D699. doi: 10.1093/nar/gkac920 PMC9825576 36263822

[44] Feldgarden M, Brover V, Haft DH, Prasad AB, Slotta DJ, Tolstoy I, et al. Erratum for Feldgarden *et al*., “Validating the AMRFinder Tool and Resistance Gene Database by Using Antimicrobial Resistance Genotype-Phenotype Correlations in a Collection of Isolates”. Antimicrob Agents Chemother. 2020;64(4):e00361-20. doi: 10.1128/AAC.00361-20 32209564 PMC7179288

[45] Bortolaia V, Kaas RS, Ruppe E, Roberts MC, Schwarz S, Cattoir V, et al. ResFinder 4.0 for predictions of phenotypes from genotypes. Journal of Antimicrobial Chemotherapy. 2020;75(12):3491–500. doi: 10.1093/jac/dkaa345 32780112 PMC7662176

[46] Letunic I, Bork P. Interactive tree of life (iTOL) v3: an online tool for the display and annotation of phylogenetic and other trees. Nucleic Acids Res. 2016;44(W1):W242-5. doi: 10.1093/nar/gkw290 27095192 PMC4987883

[47] Couvin D, Bernheim A, Toffano-Nioche C, Touchon M, Michalik J, Néron B, et al. CRISPRCasFinder, an update of CRISRFinder, includes a portable version, enhanced performance and integrates search for Cas proteins. Nucleic Acids Res. 2018;46(W1):W246–51. doi: 10.1093/nar/gky425 29790974 PMC6030898

[48] CLSI. M07: Dilution AST for aerobically grown bacteria. Clinical and Laboratory Standards Institute. 2018.

[49] CLSI (Clinical and Laboratory Standards Institute). M100: Performance standards for antimicrobial susceptibility testing. 2021. 31st Edition. 10.1128/JCM.00213-21 PMC8601225 34550809

[50] Cheng T-C, Zhang Y-L, Liu C, Xu P-Z, Gao Z-H, Xia Q-Y, et al. Identification and analysis of Toll-related genes in the domesticated silkworm, Bombyx mori. Dev Comp Immunol. 2008;32(5):464–75. doi: 10.1016/j.dci.2007.03.010 17499357

[51] Hertz CJ, Wu Q, Porter EM, Zhang YJ, Weismüller K-H, Godowski PJ, et al. Activation of Toll-like receptor 2 on human tracheobronchial epithelial cells induces the antimicrobial peptide human beta defensin-2. J Immunol. 2003;171(12):6820–6. doi: 10.4049/jimmunol.171.12.6820 14662888

[52] Scharf S, Zahlten J, Szymanski K, Hippenstiel S, Suttorp N, N’Guessan PD. Streptococcus pneumoniae induces human β-defensin-2 and -3 in human lung epithelium. Exp Lung Res. 2012;38(2):100–10. doi: 10.3109/01902148.2011.652802 22296408

[53] Kaito C, Kurokawa K, Matsumoto Y, Terao Y, Kawabata S, Hamada S, et al. Silkworm pathogenic bacteria infection model for identification of novel virulence genes. Mol Microbiol. 2005;56(4):934–44. doi: 10.1111/j.1365-2958.2005.04596.x 15853881

[54] Hsieh Y-C, Lin T-L, Chang K-Y, Huang Y-C, Chen C-J, Lin T-Y, et al. Expansion and evolution of Streptococcus pneumoniae serotype 19A ST320 clone as compared to its ancestral clone, Taiwan19F-14 (ST236). J Infect Dis. 2013;208(2):203–10. doi: 10.1093/infdis/jit145 23559465

[55] Cerenius L, Söderhäll K. The prophenoloxidase-activating system in invertebrates. Immunol Rev. 2004;198:116–26. doi: 10.1111/j.0105-2896.2004.00116.x 15199959

[56] Kaito C, Akimitsu N, Watanabe H, Sekimizu K. Silkworm larvae as an animal model of bacterial infection pathogenic to humans. Microb Pathog. 2002;32(4):183–90. doi: 10.1006/mpat.2002.0494 12079408

[57] Grousd JA, Rich HE, Alcorn JF. Host-Pathogen Interactions in Gram-Positive Bacterial Pneumonia. Clin Microbiol Rev. 2019;32(3). doi: 10.1128/cmr.00107-18 PMC6589866 31142498

[58] Zak O, Tosch W, Sande MA. Correlation of antibacterial activities of antibiotics in vitro and in animal models of infection. J Antimicrob Chemother. 1985;15 Suppl A:273–82. doi: 10.1093/jac/15.suppl_a.273 3980330

[59] Zasloff M. Antimicrobial peptides of multicellular organisms. Nature. 2002;415(6870):389–95. doi: 10.1038/415389a 11807545

[60] Eleftherianos I, Zhang W, Heryanto C, Mohamed A, Contreras G, Tettamanti G, et al. Diversity of insect antimicrobial peptides and proteins - A functional perspective: A review. Int J Biol Macromol. 2021;191:277–87. doi: 10.1016/j.ijbiomac.2021.09.082 34543628

[61] Nesa J, Sadat A, Buccini DF, Kati A, Mandal AK, Franco OL. Antimicrobial peptides from Bombyx mori: a splendid immune defense response in silkworms. RSC Adv. 2020;10(1):512–23. doi: 10.1039/c9ra06864c 35492565 PMC9047522

[62] Zhang L-J, Gallo RL. Antimicrobial peptides. Curr Biol. 2016;26(1):R14-9. doi: 10.1016/j.cub.2015.11.017 26766224

[63] Wang X-Y, Li T, Johannes M, Xu J-P, Sun X, Qin S, et al. The regulation of crecropin-A and gloverin 2 by the silkworm Toll-like gene 18 wheeler in immune response. J Invertebr Pathol. 2019;164:49–58. doi: 10.1016/j.jip.2019.04.006 31026465

[64] Ooi JY, Yagi Y, Hu X, Ip YT. The Drosophila Toll-9 activates a constitutive antimicrobial defense. EMBO Rep. 2002;3(1):82–7. doi: 10.1093/embo-reports/kvf004 11751574 PMC1083923

[65] Narbonne-Reveau K, Charroux B, Royet J. Lack of an antibacterial response defect in Drosophila Toll-9 mutant. PLoS One. 2011;6(2):e17470. doi: 10.1371/journal.pone.0017470 21386906 PMC3046252

[66] Moens L, Verhaegen J, Pierik M, Vermeire S, De Boeck K, Peetermans WE, et al. Toll-like receptor 2 and Toll-like receptor 4 polymorphisms in invasive pneumococcal disease. Microbes and Infection. 2007;9(1):15–20. doi: 10.1016/j.micinf.2006.10.002 17196867

[67] Wu S, Zhang X, Chen X, Cao P, Beerntsen BT, Ling E. BmToll9, an Arthropod conservative Toll, is likely involved in the local gut immune response in the silkworm, Bombyx mori. Dev Comp Immunol. 2010;34(2):93–6. doi: 10.1016/j.dci.2009.08.010 19723534

[68] Hayashi S, Kondo T. Development and Function of the Drosophila Tracheal System. Genetics. 2018;209(2):367–80. doi: 10.1534/genetics.117.300167 29844090 PMC5972413

[69] Dessing MC, Schouten M, Draing C, Levi M, von Aulock S, van der Poll T. Role played by Toll-like receptors 2 and 4 in lipoteichoic acid-induced lung inflammation and coagulation. J Infect Dis. 2008;197(2):245–52. doi: 10.1086/524873 18179383

[70] Theopold U, Schmidt O, Söderhäll K, Dushay MS. Coagulation in arthropods: defence, wound closure and healing. Trends Immunol. 2004;25(6):289–94. doi: 10.1016/j.it.2004.03.004 15145318

