# Correction: *Streptococcus pneumoniae* upregulates *Toll2*, *Toll9*, and *defensin* genes in *Bombyx* larvae infection model

**DOI:** 10.1371/journal.pone.0344379

**Published:** 2026-03-05

**Authors:** The PLOS One Staff

The ORCID iD for the second author is incorrect and should instead be attributed to the 11^th^ author: Muktadir S. Hossain. Please see the author’s respective ORCID iD here:

- Author Muktadir S. Hossain’s ORCID iD is: 0000-0002-7239-4577 (https://orcid.org/0000-0002-7239-4577).

The publisher apologizes for the error.
