# Supplementary material for: Streptococcus pneumoniae upregulates Toll2, Toll9, and defensin genes in Bombyx larvae infection model
Source: PLoS One. 2026 Jan 30;21(1):e0341929. doi: 10.1371/journal.pone.0341929 (PMC12857934; doi:10.1371/journal.pone.0341929)
Supplement: S1 Table — (DOCX) [file pone.0341929.s009.docx]

**S1 Table.** Biochemical tests of the *S. pneumoniae*, Spn1 strain, used in this study.

| Biochemical test/properties | Results |
| --- | --- |
| Gram staining | Positive |
| Shape | Diplococci |
| Hemolysis | Alpha |
| Catalase | Negative |
| Oxidase | Negative |
| Motility | Non-motile |
| Indole | Negative |
| Urease | Negative |
| Voges Proskauer | Negative |
| Glucose fermentation | Positive |
