# Supplementary material for: Streptococcus pneumoniae upregulates Toll2, Toll9, and defensin genes in Bombyx larvae infection model
Source: PLoS One. 2026 Jan 30;21(1):e0341929. doi: 10.1371/journal.pone.0341929 (PMC12857934; doi:10.1371/journal.pone.0341929)
Supplement: S2 Table — (DOCX) [file pone.0341929.s010.docx]

**S2 Table**: Antimicrobial resistance (AMR) profile of *S. pneumoniae,* Spn1 strain used in this study.

| Antibiotic | Sensitivity |
| --- | --- |
| Tetracycline | R |
| Ceftriaxone | S |
| Ampicillin | S |
| Imipenem | S |
| Azithromycin | R |
| Gentamycin | S |
| Penicillin | S |
| Nalidixic Acid | S |
| Ciprofloxacin | S |
| Levofloxacin | S |
| Clindamycin | S |
| Erythromycin | R |
| Meropenem | S |
| Doxycycline | S |
| Co-trimoxazole | S |
| Cefixime | S |
| Vancomycin | S |
