# Supplementary material for: Streptococcus pneumoniae upregulates Toll2, Toll9, and defensin genes in Bombyx larvae infection model
Source: PLoS One. 2026 Jan 30;21(1):e0341929. doi: 10.1371/journal.pone.0341929 (PMC12857934; doi:10.1371/journal.pone.0341929)
Supplement: S3 Table — (DOCX) [file pone.0341929.s011.docx]

**S3 Table**. Primers used for RT-qPCR in this study.

| Gene | Primers | Sequence (5′-3′) | Reference |
| --- | --- | --- | --- |
| *Bmrp49* | Forward | CAGGCGGTTCAAGGGTCAATAC | (Tsubota et al., 2014) |
|  | Reverse | TGCTGGGCTCTTTCCACGA |  |
| *BmCecropinD1* | Forward | TCAGGATCGGCTCCGTCAG | (Huang et al., 2009) |
|  | Reverse | GCGGGAAGTGCCTCTGGAA |  |
| *BmDefensinA* | Forward | AGCAAGTTTCTGTATTTCTAGTCT | (Kaneko et al., 2008) |
|  | Reverse | ACGCAAACACAAACACGA |  |
| *BmDefensinB* | Forward | GATTGGATTATCCAGGCGG | (Zhang et al., 2018) |
|  | Reverse | AACAGTATTGTTCTGATGAGAGATAG |  |
| *BmGloverin2* | Forward | GAAGATTACTCGATCAGCGG | (Geng et al., 2016) |
|  | Reverse | CTTTTCCAAAGAGGCCATCA |  |
| *BmGloverin3* | Forward | GACACGAGAATGGGAGGAG | (Ma et al., 2019) |
|  | Reverse | AAGACCCTGGTGCCGTAA |  |
| *Bmppo1* | Forward | ATGCCTCTGGACGAAG | (Huang et al., 2009) |
|  | Reverse | CAGGTCTCCCATCACG |  |
| *Bmppo2* | Forward | TTCCAGAGGCTTACTTCCC | (Huang et al., 2009) |
|  | Reverse | GTTTCTTCTCCAGCGTTCC |  |
| *BmToll2* | Forward | GAGTCCAGAGTTCGTCGACC | (This study) |
|  | Reverse | AACTCGCGTCTCCTATTCGG |  |
| *BmToll9-1* | Forward | CATGAGTCAGTGGTGCCAGT | (Liu et al., 2024a) |
|  | Reverse | GATAGTGGAGGGTCGTTGGG |  |
| *BmToll9-2* | Forward | GGTTACAAGCGAACGGTAGC | (Liu et al., 2024b ) |
|  | Reverse | CCAAATATCCGGACTGCTGC |  |
