# Supplementary material for: Streptococcus pneumoniae upregulates Toll2, Toll9, and defensin genes in Bombyx larvae infection model
Source: PLoS One. 2026 Jan 30;21(1):e0341929. doi: 10.1371/journal.pone.0341929 (PMC12857934; doi:10.1371/journal.pone.0341929)
Supplement: S4 Table — (DOCX) [file pone.0341929.s012.docx]

**Table S4**. repUS423 plasmid identified in the *S. pneumoniae*, Spn1 strain used in this study

| Plasmid | Identity | Query / Template length | Contig | Position in contig | Note | Accession number |
| --- | --- | --- | --- | --- | --- | --- |
| repUS43 | 99.83 | 1206 / 1206 | contig00001 len=285344 cov=30.5 corr=0 origname=NODE_1_length_285344_cov_30.500492 sw=shovill-spades/1.1.0 date=20240326 | 111880..113085 | CDS12738(DOp1) | [CP003584](http://www.ncbi.nlm.nih.gov/nuccore/CP003584) |
