# Supplementary material for: Streptococcus pneumoniae upregulates Toll2, Toll9, and defensin genes in Bombyx larvae infection model
Source: PLoS One. 2026 Jan 30;21(1):e0341929. doi: 10.1371/journal.pone.0341929 (PMC12857934; doi:10.1371/journal.pone.0341929)
Supplement: S6 Table — (DOCX) [file pone.0341929.s014.docx]

**S6 Table.** Summary of the antimicrobial resistance genes present in the genome of *Streptococcus pneumoniae*, Spn1 strain used in this study identified by four different databases.

| Name of the tools | Gene | Coverage (%) | Resistance of Drug |
| --- | --- | --- | --- |
| AMRfinder | *mef(A)* | 100 | Macrolide |
|  | *tet(M)* | 100 | Tetracycline |
| CARD | *patA* | 100 | Fluoroquinolone |
|  | *tetM* | 100 | Tetracycline |
|  | *RlmA(II)* | 100 | Lincosamide; Macrolide |
| NCBI | *cat-TC* | 100 | Chloramphenicol |
|  | *erm(B)* | 100 | Macrolide |
| Resfinder | *msr(D)_2* | 100 | Erythromycin; Azithromycin; Telithromycin; Quinupristin; Pristinamycin_IA; Virginiamycin_S |
|  | *tet(M)_12* | 100 | Tetracycline |
