# Supplementary material for: Streptococcus pneumoniae upregulates Toll2, Toll9, and defensin genes in Bombyx larvae infection model
Source: PLoS One. 2026 Jan 30;21(1):e0341929. doi: 10.1371/journal.pone.0341929 (PMC12857934; doi:10.1371/journal.pone.0341929)
Supplement: S7 Table — (DOCX) [file pone.0341929.s015.docx]

**S7 Table.** The CRISPR/Cas system of *S. pneumoniae*, Spn1 strain used in this study.

| Element | CRISPR Id/Cas Type | Start | End | Spacer /  Gene | Repeat consensus /  cas genes |
| --- | --- | --- | --- | --- | --- |
| CRISPR | - | 22880 | 22964 | 1 | CTTTTTTTGAAACGTTTCATTTTT |
| Cas cluster | CAS | 15076 | 16419 | 2 | cas3_TypeI, cas3_TypeI |
